# Burden of Childhood Malnutrition: A Roadmap of Global and European Policies Promoting Healthy Nutrition for Infants and Young Children

**DOI:** 10.3390/children9081179

**Published:** 2022-08-06

**Authors:** Marianthi Sotiraki, Aggeliki Malliou, Ntaniela Tachirai, Nikoletta Kellari, Maria G. Grammatikopoulou, Theodoros N. Sergentanis, Tonia Vassilakou

**Affiliations:** 1 MSc Programme in Public Health, Department of Public Health Policy, School of Public Health, University of West Attica, Athens University Campus, 196 Alexandras Avenue, 11521 Athens, Greece; 2 Department of Rheumatology and Clinical Immunology, University General Hospital of Larissa, Faculty of Medicine, School of Health Sciences, University of Thessaly, 41221 Larissa, Greece

**Keywords:** nutrition policies, breastfeeding policies, childhood malnutrition, infants, young children

## Abstract

Childhood malnutrition is a global epidemic with significant public health ramifications. The alarming increase in childhood obesity rates, in conjunction with the COVID-19 pandemic, pose major challenges. The present review aims to critically discuss policies and action plans promoting healthy nutrition among infants and children, globally. Since the Convention on the Rights of the Child in 1989 and the joint consortium held by the World Health Organization (WHO) and the United Nations Children’s Fund (UNICEF) that led to the “Ten Steps to Successful Breastfeeding”, several policymakers and scientific societies have produced relevant reports. Today, the WHO and UNICEF remain the key players on the field, elaborating the guidelines shaped by international expert teams over time, but we still have a long way to go before assuring the health of our children.

## 1. Introduction

At a time when nutrition research is evolving, the challenges associated with food insecurity and malnutrition appear to be threatening. Although the access to food and resources has become more effortless in high-income countries, food deprivation remains the core problem in the developing world [1]. Furthermore, scientists underline the alarming increase in malnutrition rates globally, and, due to its severe impact on infants and children, numerous studies have addressed its different forms [1].

The literature on the subject brings together several definitions. According to World Health Organization (WHO), the term “malnutrition” is used to describe the imbalanced nutrition, including either deficit or excess. Nutritional excesses manifest in overweight and obesity. Undernutrition refers to the inadequate nutrient intake and encompasses wasting, stunting, underweight, and micronutrient deficiencies [2]. The simultaneous manifestation of both undernutrition and overweight/obesity—or diet-related non-communicable diseases (NCDs) often referred to as “the double burden of malnutrition”, is an evolving major problem in developing countries [3]. Some authors, though, refer to “the triple burden of malnutrition”, which includes the three manifestations of malnutrition, namely hidden hunger, increased consumption of ultra-processed foods and obesity. Hidden hunger denotes a chronic micronutrient deficiency and applies to both high-income and low-and middle-income countries [4]. Hunger is the condition when a person’s food intake is inadequate to cover his needs, forcing him to seek more food; “undernourishment” can also be used instead of “hunger” [5]. On the other hand, food insecurity is often the common cause of hunger and malnutrition, and describes the limited access to adequate food for a healthy life [6].

The burden of malnutrition signals a worldwide epidemic that poses public health challenges. Africa and Asia bear the greatest share of all forms of malnutrition [7]. Undernutrition accounts for 45% of the mortality in children under the age of 5 years [1,8]. According to the updated edition on childhood malnutrition estimates, published by WHO, United Nations Children’s Fund (UNICEF) and the World Bank Group in March 2020, in the year 2019, 144 million children under 5 suffered from stunting (low height-for-age), 47 million were wasted (low weight-for-height) and 38.3 million were overweight [9].

Despite the variety of policies and actions that have been implemented, childhood obesity continues to rise [1]. Results of the 2017–2018 National Health and Nutrition Examination Survey (NHANES) [10] indicate that 13.4% of the children aged 2–5 and 1/5 (20.3%) of those aged 6–11-years old in the USA were obese. These rates have been substantially increased since the year 1970, when the first surveys were conducted [10]. In the European region, the most recent report by the WHO European Childhood Obesity Surveillance Initiative (COSI) revealed that 29% of the boys and 27% of the girls aged 7–9 years old were overweight, including obesity [11]. Moreover, these rates were unequally distributed throughout the European region [11]. The Mediterranean countries experience the greatest burden (between 38% and 43%), whereas in Tajikistan, Kyrgyzstan and Turkmenistan the prevalence of overweight was low, ranging between 5% and 11%. Furthermore, the COVID-19 pandemic induced further increases in the prevalence of childhood obesity [12], as reported by the Centers of Disease Control and Prevention (CDC) [13].

Developmental delay, growth and cognitive impairment, susceptibility to infections have all been associated with undernutrition [14]. On the other hand, childhood overweight and obesity is a major risk factor for non-communicable diseases (NCDs) [15]. Other consequences include mental health problems, discrimination and low academic performance [16]. Moreover, obese children are at increased risk of becoming overweight or obese adults, a fact that raises implications for their good health and well-being across life course [17,18].

Paradoxically, children who have suffered from undernutrition in early life are at greater risk of developing overweight and obesity over time, compared to the children with normal birth weight and secular growth [19]. This highlights the importance of a healthy diet in the intrauterine and postnatal life. Many studies have indicated that neonatal adiposity (fat mass percentage) plays a key role in developing overweight and obesity in early childhood and have suggested early interventions regarding maternal diet during pregnancy [20]. The Barker hypothesis claims that fetus and infant malnutrition leads to increased susceptibility to the metabolic syndrome [21]. The 1000-days window refers to the first thousand days of life, that last from conception to the end of the second year of life and represents a unique period with great impact on children’s long-term health, growth, and neurodevelopment across lifespan in an irreversible way [22].

The short- and long-term benefits of breastfeeding have been well established during the past decades. Human milk and its nutritional components have been associated with various health outcomes, such as healthy gut microbiome, good gastrointestinal function, neurobehavioral development and healthy immune system [23,24]. Furthermore, breastfeeding initiation has been linked with reduced infant mortality, according to the results from a recent longitudinal study in the United States [23]. It is estimated that over 820,000 infant deaths could be prevented through optimal breastfeeding practices [24]. Promotion of exclusive breastfeeding, at least during the first six months of life, is widely recognized as one of the most effective policies to ensure adequate and healthy nutrition and short- and longterm health for all infants and mothers. However, only 40% of babies are exclusively breastfed for the first six months [25].

The objective of the present narrative review is to summarize most of the published policies by international organizations and interested parties over time, aiming in promoting healthy nutrition among infants and young children, and to critically highlight the existing gaps and future challenges.

## 2. Materials and Methods

Policies aiming to present policies promoting healthy nutrition among infants and young children were initially retrieved through electronic searches of the websites of the main organizations responsible for policy making among children, including:

- Word Health Organization (WHO) (https://www.who.int/) (accessed on 26 March 2022)
- Centers for Disease Control and Prevention (CDC) (https://www.cdc.gov/) (accessed on 26 March 2022)
- United Nations Children’s Fund (UNICEF) (https://www.unicef.org/) (accessed on 26 March 2022)
- Word Health Organization Regional Office for Europe (WHO/Europe) (https://www.euro.who.int/en) (accessed on 26 March 2022)
- European Centre for Disease Prevention and Control (ECDC) (https://www.ecdc.europa.eu/en) (accessed on 26 March 2022)
- U. S. Department of Agriculture (USDA) (https://www.usda.gov/) (accessed on 26 March 2022)

Our initial search among WHO statements and reports was headed to five, main WHO Teams, the “Child Health and Development Unit”, the “Maternal Health Unit”, the Department of “Maternal, Newborn, Child and Adolescent Health and Ageing”, the Department of “Newborn Health”, and the “Nutrition and Food Safety” Unit. The list of Networks, Committees, Advisory Groups and Taskforces was concurrently investigated for endorsed groups and guidelines or technical packages, which were retrieved. Information from policy statements and guidelines were extracted. The WHO Regional Office for Europe citing contextual material under the topic “Child and adolescent health” was also examined.

Similar publications were sought on the UNICEF’s website, many of them co-authored by WHO, as well.

The American Academy of Pediatrics relevant guidelines were also searched, in particular with regard to breastfeeding, complementary feeding and introduction of solid foods, prevention of malnutrition and sustainment of healthy nutrition during childhood. The field named “Childhood Overweight and Obesity” on the CDC website was also carefully examined. USDA’s website, presenting all governed programs aiming to obtain access to healthy food for all people in the USA, was also included in the search.

A parallel search in electronic databases was conducted, including MEDLINE, Google Scholar and the Cochrane Library. Reviews and guideline statements were assessed for their relevance, either focusing on existing guidance or introducing new insights on the topic, providing though important information to this review. We also searched the UpToDate resource by Wolters Kluwer in order to retrieve evidence-based reviews on the topic. The following keywords were used in all search engines, either separately or composing a search strategy, where applicable: nutrition policy (MeSH term)/strategy/action plan, malnutrition (MeSH term), infant and child feeding, breastfeeding (MeSH term), dietary recommendations.

Manual searching in the reference lists of all studies yielded through the aforementioned strategy followed, aiming to identify any additional relevant reports. Suggested Society Guidelines Links were also verified.

Eligible documents for our study were reports, action plans and policies published in English by endorsed organizations, which focused either entirely or partly to infants and children, with respect to healthy nutrition promotion. Publications that did not mention specific policies towards children or were restricted to targets regarding adolescence or pregnancy, as well as publications referring to nutritional interventions or policy implementation were not included in our study. No restrictions were applied on the publication date. Literature was searched through March 2022.

## 3. Results

Policy strategies and action plans upon children’s healthy eating can be presented into five distinct categories: Breastfeeding, complementary feeding and young child feeding promotion; severe malnutrition prevention and management; prevention of childhood overweight and obesity; children’s healthy nutrition as a goal in broader plans; strategies and action plans in the European region.

### 3.1. Breastfeeding, Complementary Feeding and Young Child Feeding Promotion

In 1989, WHO and UNICEF developed the first detailed statement for the protection and promotion of breastfeeding, with the awe-inspiring goal “to re-establish a breastfeeding culture”. In a decade when breastfeeding was considered old-fashioned, due to the pronounced promotion of bottle-feeding, the attempt to minimize the use of the so-called “modern technologies” would have been vain if the global authorities had not asked for commitment. Specific actions, including policy-making for maternity facilities, as well as health professionals’ training were strongly suggested, on the basis of encouraging people to embrace the use of human milk. For this purpose, the “Ten Steps to Successful Breastfeeding” were announced and, since then, all upcoming strategies would refer to this guidance [26].

A year later, WHO and UNICEF co-organized a conference on “Breastfeeding in the 1990s: A Global Initiative” in Spedale degli Innocenti, Florence, Italy, which concluded with the “Innocenti Declaration” [27]. This short statement disseminated the knowledge and promoted public understanding about the need to reinforce the “breastfeeding culture”. The experts proposed that governments could be checked for progress by 1995 towards specific operational targets, regarding the implementation of the Ten Steps through legislation and policies that would protect, promote and support breastfeeding. Shortly after that, a confirmatory guidance for the importance and duration of exclusive breastfeeding was declared by the World Summit on Children and the contextual report “World Declaration on the survival, protection and development of children and Plan of Action for Implementing the World Declaration on the survival, protection and development of children in the 1990s” [28].

Despite the efforts towards the establishment of breastfeeding, the insufficiency of policy frameworks on imposing measures including maternity facilities gave birth to the launch of the “Baby-Friendly Hospital Initiative” by WHO and UNICEF in 1991 [29]. Hospitals that fulfilled specific criteria and met the standards for implementing the Innocenti Declaration could be certified as “Baby-Friendly Hospitals”, ascertaining for their services and enhancing their prestige. Innocenti Declaration and BFHI would be the cornerstones of policies and a common point of reference of all decades.

At that time, significant efforts were made throughout the world leading to the foundation of prominent organizations and networks dedicated to the accomplishment of the mission of the Innocenti Declaration. World Alliance for Breastfeeding Action (WABA) is a network for the protection, promotion and support of breastfeeding, that has been coordinating meetings, courses, strategies and campaigns worldwide since 1992. At that time, a campaign of special interest was launched, the “World Breastfeeding Day” (WBD), indicating the initiation of celebrations and activities to support mothers holistically. Recognized by UNICEF later and endorsed by the 71st World Health Assembly (WHA), this campaign remains at the forefront of actions and since 2016 it has been aligned to the Sustainable Development Goals (SDGs), creating the WBD-SDGs Campaign [30].

In 2002, WHO and UNICEF launched the Global Strategy for Infant and Young Child Feeding, based on the Innocenti Declaration and the BFHI, and the project was endorsed by the 55th WHA. Declaring the importance of the promotion of breastfeeding, the Strategy reaffirmed the four Innocenti targets and integrated the new guidelines for optimal duration of exclusive breastfeeding that had been published one year before by WHO. The use of human milk for up to the 6th month of life in an exclusive way and the continuation of breastfeeding for at least two years, along with complementary feeding, was the key point of the guidance [31]. Moreover, the importance of best feeding practices for both babies and mothers was articulated, in the context of promoting mother-friendly facilities too. On the other hand, the arising pandemic of the human immunodeficiency virus (HIV), that dominated health systems worldwide, necessitated immediate actions and explicit instructions for infected mothers, with regard to the prevention of mother-to-child transmission. The strategy called for cooperation between governments, organizations and individuals and added five new targets concerning the reconstruction of health policies. Raising public awareness and forming innovational systems consisted the main objectives of the action [32].

Meanwhile, a joint statement issued by Pan American Health Organization (PAHO) and WHO in 2003, the “Guiding Principles for complementary feeding of the breastfed child”, provided detailed information about the introduction of solid foods at the 6th month after birth and the nutritive practices through childhood, as well as about supplementary intakes and fortified products [33]. That was the first policy statement underlining the incalculable value of healthy nutrition in the crucial period through the second year of life, which determines child’s health, a hypothesis dated back in 1990s and expressed by the British epidemiologist David Barker [21].

The celebration of the 15th Anniversary of the Innocenti Declaration on the Protection, Promotion and Support of Breastfeeding in 1990 gathered all interested parties in Convitto della Calza, Florence, Italy, and a featured, extensive report of utmost importance was written by the experts [34]. The event resulted in the revision of the Innocenti Declaration and gave birth to “The Innocenti Declaration 2005 on Infant and Young Child Feeding”, with reference to the achievement of the Millennium Development Goals (MDGs) and the impediments of meeting the targets which had been set in the policies aforementioned. The report required advocacy from governments, non-governmental organizations and institutions, and demanded full compliance from milk substitutes industries [35]. The 59th WHA in 2006 unanimously welcomed the updated Innocenti Declaration through a special report to the infant and young child nutrition in its 59.21 resolution.

**Figure 1:**
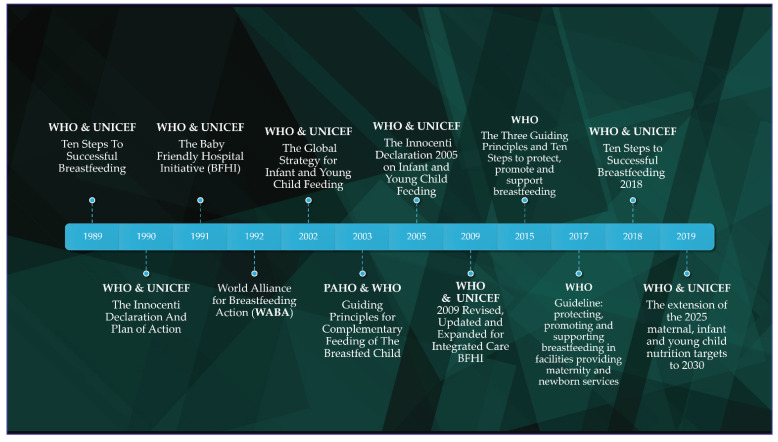
Timeline of the international strategies for improving infant and young child feeding.

In 2009 the BFHI originators assembled again in the view of the necessity to integrate the new nutrition guidelines, as well as manage the HIV burden on mothers. Thousands of hospitals had been certified formerly throughout the world, but the unprincipled marketing of breast-milk substitutes still prevailed. The 2009 Revised, Updated and Expanded for Integrated Care BFHI package consisted of five sections, methodically structured, and included comprehensive information and guidance to hospitals, facilities, administrators and policymakers, along with a 20-h course for maternity staff [36]. In March 2015 the BFHI was expanded to neonatal wards, addressing the challenges of prematurity and hospitalization. Based on the earliest research findings about the vital role of breastfeeding in reducing neonatal mortality, the Three Guiding Principles and Ten Steps to protect, promote and support breastfeeding redefined the goals [37]. 30 years after the first attempt to establish the “breastfeeding culture”, the BFHI received a revision that focused on enhancing the appeal of the initiative and on ensuring that all maternity facilities adopt the policy. Based on the previously developed Guideline for protecting, promoting and supporting breastfeeding in facilities providing maternity and newborn services by WHO [38], 2017, the revised Ten Steps to Successful Breastfeeding 2018, reviewed in an extensive way in the relative document, applied to almost all maternal and neonatal services [39]. The 5th target refers to the exclusive breastfeeding rates and an increase to 50% by 2025 was demanded. An update to the scope of work evolved recently, redefining the target by an increase to 70% by 2030. The extension and revision of all of the aforementioned nutrition targets was reported in the relevant WHO/UNICEF discussion paper that was published in June 2019 [40]. A comprehensive timeline of the international strategies that promote breastfeeding and optimal eating habits for children is shown in Figure 1.

Many other actions have been developed in the field of establishing healthy nutrition practices with respect to postnatal care. Since 1991, WHO supervised the projects to attain consensus upon the assessment of infant feeding [41]. “The Indicators for assessing breastfeeding practices” was the first document, followed by the revised one in 2008, “Indicators for assessing infant and young child feeding practices”. Since then, frequent reports were published in order to appraise the situation within countries, and the proportion of infants 0–5 months of age (0 to <6 months) who exclusively breastfed was used as the main indicator to be measured [42]. In 2021, WHO and UNICEF updated the document, aiming to provide assessment, targeting interventions and monitoring the progress towards mother’s support to breastfeed. Breastfeeding, complementary feeding and other indicators were described in detail, introducing more precise methods for the evaluation of practices [43].

Aiming to an integrated and up-to-date supervision of newborn’s health, WHO expert teams have periodically published guiding issues concerning newborn care and infant feeding. Since 1993, many documents aimed to address this need, such as Breastfeeding counselling: a training course [44], Infant and young child feeding counselling: an integrated course [45], WHO recommendations on postnatal care of the mother and newborn [46], Early Essential Newborn Care-Clinical practice pocket guide [47], Infant and young child feeding: model chapter for textbooks for medical students and allied health professionals [48]. The last one gave full guidance to medical students and physicians, along with a detailed evidence base, emphasizing an unambiguous lack in health professional’s training and knowledge of optimal infant feeding practices. The WHO Essential Newborn Care (ENC) program was initiated aiming to reduce neonatal mortality and morbidity and recommending early breastfeeding induction after birth [49].

Another important field of development that caused divergence between the stakeholders was the legislation and policy covering the breast-milk substitutes trade. The main action to this direction dated one decade before the Innocenti Declaration, when WHO and UNICEF cosponsored a meeting that accounted for the creation of the International Code of Marketing of Breast-milk Substitutes, a code that contributed to the restriction of the aggressive marketing by the commercial infant formula companies [50]. A matter of conflict emerged though, regarding the free access to substitutes by maternity wards in cases of poverty or debilitating conditions, as many violations were reported; frequently, mothers capable of breastfeeding were encouraged by personnel to the “friendly-use” of substitutes, alleging difficulties [51]. In the context of expanding the implementation of the code, UNICEF and other organizations convened meetings and workshops providing guiding material for law formulations.

### 3.2. Severe Malnutrition Prevention and Management

Severe malnutrition and deprivation have also been fields of intensive research. Chronic and acute malnutrition are the clinical manifestations of the disease. Stunting signs for chronic malnutrition are often present in children with acute malnutrition. In 2009, through a joint statement, WHO and UNICEF published directions on the evaluation and assessment of the disease [52]. Several action plans have addressed the nutritional needs of children in low-and middle-income countries, as it is presented in Table 1.

Two guiding reports were issued by WHO in 1999 and 2000 respectively, in the context of severe, acute malnutrition management by physicians [53,54]. The Guideline: Updates on the management of severe acute malnutrition in infants and children presented the updated evidence regarding the recommendations for the treatment of severe acute malnutrition [55]. A joint statement by WHO, UNICEF, World Food Programme (WFP) and United Nations Standing Committee on Nutrition (UNSCN) in 2007 reflected the significance of coordinated efforts with community support towards the prevention of acute malnutrition [56]. In 2003, WHO recommended specific steps to increase breastfeeding rates in the developing WHO regions, considering the definite proof of its beneficial impact on childhood mortality [57].

### 3.3. Prevention of Childhood Overweight and Obesity

Childhood obesity is a common major public health problem in both developed and developing countries [1]. Various policies address the topic in the context of broader plans that either refer to early childhood healthy nutrition promotion or, generally, deal with the burden of NCDs. Table 2 presents two concrete initiatives with special interest to the prevention of childhood overweight and obesity.

Aiming to provide a comprehensive framework for action, WHO established the Commission on Ending Childhood Obesity in 2014. Two years later, in 2016, the contextual report of it, by WHO, presented the six focus areas of specific recommendations on tackling the burden of obesity [58]. The restriction in the use of unhealthy foods and sugar-sweetened beverages by children and the promotion of healthy nutrition behaviors were highlighted by the experts in the first focus area and were further analyzed into detailed guidelines. The promotion of physical activity was the main approach of the second area, declaring the necessity of reducing sedentary behaviors in children and adolescents, which are associated with obesity and other metabolic disorders. The third area focused on the importance of comprehensive counselling in the preconception and antenatal period, aiming to the long-term benefits in children’s optimal weight and good health. A detailed matrix of steps regarding the guidance on healthy diet, sleep and physical activity for infants’ and toddlers’ caregivers was presented in the fourth area, whereas the fifth one focused on contextual programs in school environments. In the last area, a holistic approach was discussed, in respect to the management of obese children and young people through family-based, multicomponent and updated services.

In 2017 WHO published a comprehensive guidance for primary health care facilities which incorporated the established recommendations and introduced new ones, regarding the prevention of childhood overweight and obesity [59]. Detailed directions were given, regarding the anthropometric and nutritional assessment of infants and children and the management of primary health care pediatric patients with acute and chronic malnutrition, overweight and obesity.

### 3.4. Children’s Healthy Nutrition as a Goal in Broader Plans

At the dawn of the new millennium, a concerted action motivated by the extreme poverty rates [60] and the aggravating global climate situation [61] resulted in the composition of an inspired contract; the Millennium Development Goals (MDGs). Children’s nutrition and optimal feeding in order to grow into their full potential was the main pillar of the 1st goal “Eradicate extreme poverty and hunger” [62].

An influential movement was initiated in 2010 by Ban Ki-moon, under the name of “Every Woman Every Child”, as a part of the “Global Strategy for Women’s and Children’s Health”. The strategy aimed to speed up the implementation of MDGs [63].

In 2015, the United Nations presented the appraisal of the work on MDGs and introduced the new vision of the “2030 Agenda for Sustainable Development”. The eight MDGs evolved into the 17 Sustainable Development Goals (SDGs). Significant goals as “Zero Hunger” and “Good Health and Well-Being” demand special efforts, providing orientation and operational targets to be achieved. Prevalence of stunting and malnutrition imply progress indicators and important questions that have to be addressed through 2030 [64]. Food insecurity, as part of the 2nd Goal, is a growing challenge for most countries; FAO introduced the Food Insecurity Experience Scale (FIES) as a tool for food insecurity assessment [65].

The “Global Strategy for Women’s, Children’s, and Adolescents’ Health (2016–2030)” advanced to its new mission to incorporate the SDGs into Ban Ki-moon’s initial ambition. Three major perspectives were performed, Survive-Thrive-Transform, and Thrive was the objective that includes the actions against malnutrition, as far as mothers, infants and young children are concerned [66]. With the prospect of sustainability and equity in health services, the Strategy broadened the policies into adolescents’ care and, in celebrating the 10th anniversary since 2010, the 2020 progress report on the” Every Woman Every Child Global Strategy for Women’s, Children’s and Adolescents’ Health” (2016–2030) redefined the movement in terms of the COVID-19 pandemic. *Rise, refocus and recover* was the new motto, with special attention to the first of six focus areas, namely the early childhood development, that underlined the key-role of optimal feeding [67].

At the same time, a policy framework of great value was introduced in the 65th WHA in 2012, in the context of the United Nations Decade of Action on Nutrition 2016–2025 [68], proposing specific targets for implementing universally the infant and young child nutrition guidelines through 2025 [69]. Experts suggested a six-target plan with five actions aiming to achieve adherence to the framework. The 1st target directed towards the reduction of stunted children under 5 by 40%, whereas the 4th stated that the increase in the number of overweight children should come to an end, being the first statement to indicate the so-called double burden of undernutrition and overweight.

WHO’s Global School Health Initiative originated as the first global effort to establish optimal school conditions in 1995 [70]. The vision was to embrace needs of all children with future perspective of health and well-being. The so-called “Health Promoting Schools” have been still working on forming their environment to control the determinants of health. The sector of nutrition remained a focus target.

**Figure 2:**
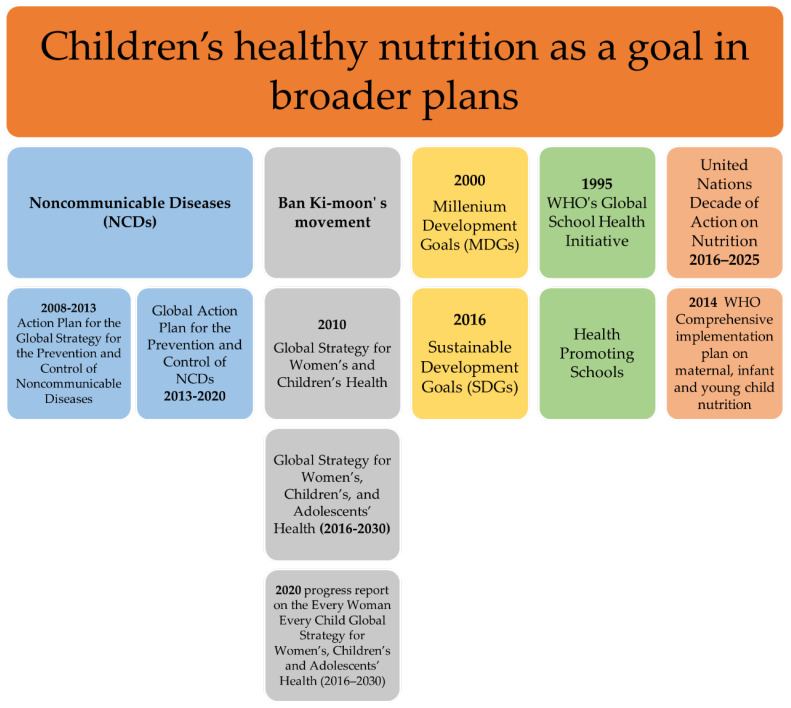
Children’s healthy nutrition as a goal in broader plans.

Another attempt to promote healthy nutrition practices was made through creating intervention plans to reduce modifiable risk factors for NCDs. The term refers to cancers, cardiovascular disease, diabetes and chronic lung illnesses. Common risk factors include tobacco use, harmful alcohol intake, physical inactivity and the adoption of unhealthy dietary patterns. In the year 2000, stakeholders welcomed the first WHO resolution for the prevention and control of NCDs, providing general guidance on the topic, without however, mentioning the needs of children [71]. The 2008–2013 Action Plan for the Global Strategy for the Prevention and Control of Noncommunicable Diseases, published by WHO, provided recommendations for exclusive breastfeeding during the first six months of life and stated for healthier composition of foods, concerning children [72]. The suggested goals were reaffirmed by the updated Global Action Plan for the Prevention and Control of NCDs 2013–2020 and current actions have been developed in the context of the “2030 Agenda for Sustainable Development” [64,73]. Figure 2 summarizes the aforementioned broader plans.

### 3.5. Strategies and Action Plans in the European Region

International nutrition policies tried to highlight the significance of an integrated approach in the promotion of children’s healthy nutrition; similar actions and policy frameworks have appeared over time in the European region as well. David Byrne, the European Commissioner for Health and Consumer Protection, at the EU Conference on Promotion of Breastfeeding in Europe on the 18th of June 2004, presented the Protection, promotion and support of breastfeeding in Europe: a blueprint for action. That was the first European action plan in the field of breastfeeding promotion and support, which had been developed two years earlier [74]. In accordance with the MDGs, the WHO regional office for Europe in 2005, developed the European strategy for child and adolescent health and development, stating the importance of the early years of life in establishing optimal nutrition practices [75]. The rising rates of childhood obesity in the following years led to the launch of the EU Action Plan on Childhood Obesity 2014–2020 in 2014, which aimed to suspend the accelerating increase of the problem among children and young people aged 0–18 years [76]. A parallel strategy was developed one year later by WHO, the European Food and Nutrition Action Plan 2015–2020, aiming to combat the preventable diet-related NCDs, obesity, and all other forms of malnutrition [77]. There had been two previous similar documents, The First action plan for food and nutrition policy: WHO European Region 2000–2005 and the WHO EUROPEAN Action Plan for Food and Nutrition Policy 2007–2012 that had tried to provide an initial framework for children’s nutrition [78,79].

An inspired program was the Schools for Health in Europe (SHE) Network Foundation, formerly named European Network of Health Promoting Schools (ENHPS), in 1992 [80]. One of the specified target points was healthy nutrition promotion and practices through schools. The SHE has continued working on advancing the educational role of schools into a holistic approach to students’ needs and for this purpose has provided support and useful material for national implementation [81]. The Healthy Eating and Physical activity in Schools (HEPS) project is a European project linked with the SHE network, aiming to offer guidance for school policy development on healthy eating and physical activity in the European region. The HEPS toolkit consists of six documents that direct to help EU member states develop their own policies [82]. Furthermore, the vision of the Best-ReMaP project 2020–2023, entitled “Healthy food for a Healthy Future” is a European joint action aiming to exchange nutrition policies and control the marketing of food and beverages to children [83].

**Figure 3:**
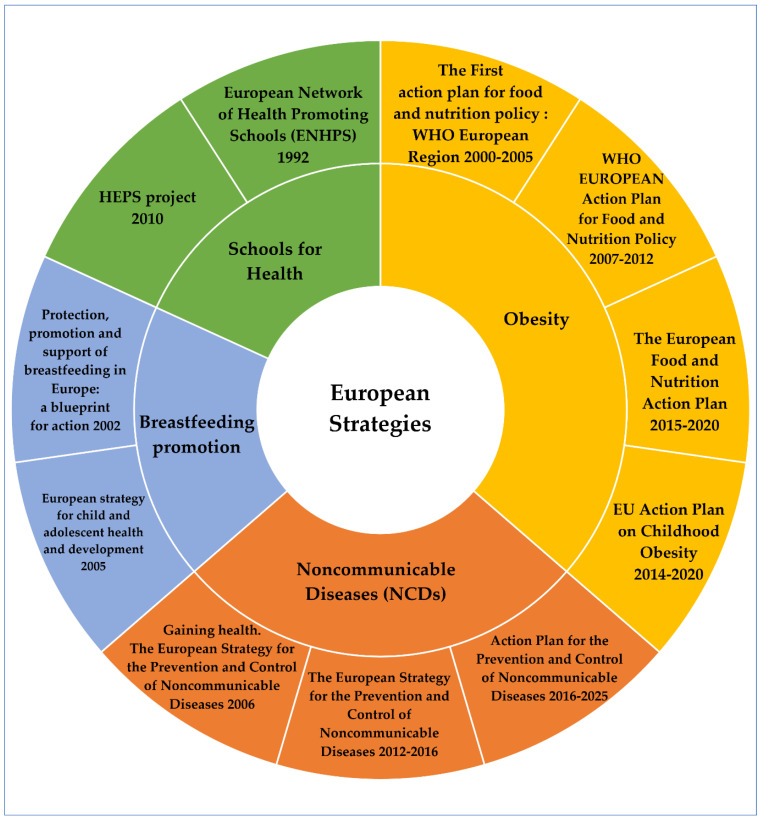
A matrix of nutrition policies targeting children in the European region.

The fight against NCDs entered the European Agenda in 2006, in compliance with the corresponding international action plans. A report entitled Gaining health. The European Strategy for the Prevention and Control of Noncommunicable Diseases and the following resolution of the Regional Committee was the first step, with special mention to reducing levels of added salt, fat and sugars in children’s nutrition [84,85]. The European Strategy for the Prevention and Control of Noncommunicable Diseases 2012–2016 and the following action plan for its implementation focused again on the necessity to control the marketing of processed food aimed at children [86]. The current Action Plan for the Prevention and Control of Noncommunicable Diseases in the WHO European Region 2016–2025 set as a priority area the food product reformulation and improvement, among others, but the initiators called for caution about the adequate iodine intake by children [87]. Figure 3 details the European nutrition strategies for healthy children without any malnutrition form, in a graphical manner.

Parallel actions that contributed to reach a consensus regarding the optimal growth and subsequently the best feeding guidance was the development of growth charts, applicable to milestone ages. The first attempt was dated in early 1900s, but it was the 1977 National Center for Health Statistics (NCHS) Growth Charts that found wide acceptance. WHO and CDC developed in 2002 the Growth Charts for the United States and this material would henceforth be incorporated into most national guidelines for the assessment of child health and development and the growth screening [88]. The International Obesity Task Force (IOTF), as part of the International Association for the Study of Obesity (IASO), aiming to the prevention of obesity, developed new cut-offs for defining overweight and obesity, which are used in several countries [89,90].

## 4. Discussion

The concept of establishing the principles of healthy nutrition on pediatrics arose in late 80’s. It is only then, that the international stakeholders decided to endorse a policy, calling for action in the field of newborns’ feeding, inspired by the pronouncement about the infants’ rights to adequate nutritious foods signified in 1989 Convention on the Rights of the Child [91]. Henceforth, the aforementioned policies have been widely accepted and several national strategies and action plans have been developed, incorporating childhood nutritional guidelines, and introducing new perspectives. However, the need for further implementation, evaluation and critical appraisal of the existing policies should be acknowledged.

Aiming to elucidate the factors leading to childhood malnutrition, WHO members periodically assemble over time, issuing reports and statements which subsequently are adopted by the WHO Regional Office for Europe. According to the WHA 62.14 document, inequities in children’s health demand actions on the social determinants of health [92]. Extreme poverty, economic instability and urbanization modify health outcomes and hamper children’s way to flourish [93]. The heads of the WHO Regional office for Europe declared that “children’s obesity is the clearest demonstration of the strength of environmental influences and the failure of the traditional prevention strategies based only on health promotion” [94]. The main documents with reference to childhood overweight and obesity [58,59,67,69,70,72,73,76,77,81,85,86,87,90], published by international and European organizations, are shown in Table 3.

The WHO Department of Maternal, Newborn, Child and Adolescent Health and Ageing (MCA) and the Department of Sexual and Reproductive Health and Research (SRH) periodically conduct the global Sexual, reproductive, maternal, newborn, child and adolescent health (SRMNCAH) policy surveys to assess implementation of guidelines [95]. The fifth SRMNCAH survey covered 16 national policy areas, many of them including nutrition-associated sectors. The results, published in 2020, indicated that, as far as malnutrition is concerned, 75% of countries have responded with policies or guidelines. The estimates were even worse regarding actions towards overweight and obesity, as only 59% of them reported contextual policies. Concerns arose, though, reflecting the inconsistency between plans, interventions and achievements. Moreover, authorities highlighted the difficulty in retrieving data, since few countries have developed appropriate monitoring systems.

A more comprehensive approach to the burden of childhood obesity reveals the great impact on various sectors of daily life. The negative psychosocial effects mainly sustain a vicious cycle of unhealthy dietary habits and sedentary behavior [16]. As stated by the initiatives, efforts should be directed to actions that address both the short-term value of attaining a healthy profile, free of metabolic risks and psychological imbalance, and the long-term sequels of obesity on next generations [58].

The COVID-19 pandemic posed an unexpected challenge and its aggravating role has now become apparent in the literature. Since an increase in all forms of malnutrition is expected, re-strategized actions should be developed for the prevention of undernutrition both in developed and developing countries. The alarming increase of food insecurity seems to be threatening, impeding progress and demanding double efforts [96]. The coexistence of this double burden, appearing as a syndemic, reveals the inadequate and ineffective existing health policies and calls for remodeling [97]. A wide range of suggested policy actions, from community interventions and counselling programs to government enforcement to support families, broaden globally the targets [98].

To our knowledge, this is the first review that brings together the major policy strategies, issued by authorized international organizations, providing guidance on implementing best dietary practices among infants and young children. Limitations in methodology, though, should be taken into account, as this is not a systematic review, whereas the wideness of the theme, as well as the considerable geographic diversity of WHO regions should be acknowledged. Due to the fact that this study is not a systematic review, it was not possible to present a flow chart with the successive steps of document selection. Moreover, the present review does not pertain to the clinical setting but to the public health field; therefore, clinical interventions, such as pharmacological therapy or bariatric surgery rarely applied in selected cases, do not fall into the scope of this review.

## 5. Conclusions

Since 2003, when the executive heads of WHO and UNICEF announced that “There can be no delay in applying the accumulated knowledge and experience to help make our world a truly fit environment where all children can thrive and achieve their full potential”, remarkable efforts have been made to address the problem of childhood malnutrition. Current health estimates, however, show significant delays on the progress. The children of the world hope for food security and equity in resources, indicating that considerable steps are pending.

## Figures and Tables

**Table 1:** Acute malnutrition management policies.

Title of Policy	Publication Year	Organization	Approach	Target Population
Management of severe malnutrition: a manual for physicians and other senior health workers.	1999	WHO	Management	LMIC
Management of the child with a serious infection or severe malnutrition: guidelines for care at the first-referral level in developing countries.	2000	WHO	Management	LMIC
Community-based strategies for breastfeeding promotion and support in developing countries	2003	WHO	Prevention	LMIC
Community-based management of severe acute malnutrition: a joint statement by the WHO, the WFP, the UNSCN and the UNICEF	2007	WHO/UNICEF/WFP/UNSCN	Prevention	International
WHO Child growth standards and the identification of severe acute malnutrition in infants and children	2009	WHO & UNICEF	Evaluation and assessment	International
Guideline: Updates on the management of severe acute malnutrition in infants and children	2013	WHO	Management	International

LMIC, low-medium income countries; UNICEF, United Nations Children’s Fund; UNSCN, United Nations Standing Committee on Nutrition; WHO, World Health Organization; WFP, World Food Programme.

**Table 2:** Policies for prevention of childhood overweight and obesity.

Policies	Publication Year	Organization	Focus Areas
Report of the commission on ending childhood obesity	2016	WHO	Promote intake of healthy foods
			Promote physical activity
			Preconception and pregnancy care
			Early childhood diet and physical activity
			Health, nutrition and physical activity for school-age children
			Weight management.
Guideline: assessing and managing children at primary health-care facilities to prevent overweight and obesity in the context of the double burden of malnutrition	2017	WHO	Anthropometric assessment
			Management of acute or chronic malnutrition
			Care of overweight or obese children
			Care of overweight or obese children

WHO, World Health Organization.

**Table 3:** Documents with reference to childhood overweight and obesity.

Policies	Publication Year	Organization	Target Population
Health Promoting Schools	NA	WHO	International
Extended International (IOTF) Body Mass Index Cut-Offs for Thinness, Overweight and Obesity in Children	NA	IOTF	International
Gaining health. The European Strategy for the Prevention and Control of Noncommunicable Diseases	2006	WHO Europe	Europe
2008–2013 Action Plan for the Global Strategy for the Prevention and Control of Noncommunicable Diseases	2009	WHO	International
Healthy Eating and Physical activity in Schools (HEPS) project	2010	The Schools for Health in Europe network (SHE)	Europe
Action plan for implementation of the European Strategy for the Prevention and Control of Noncommunicable Diseases 2012−2016	2012	WHO Europe	Europe
Global Action Plan for the Prevention and Control of NCDs 2013–2020	2013	WHO	International
WHO Comprehensive implementation plan on maternal, infant and young child nutrition	2014	WHO	International
EU Action Plan on Childhood Obesity 2014–2020	2014	European Commission	Europe
European Food and Nutrition Action Plan 2015–2020	2015	WHO Europe	Europe
Report of the commission on ending childhood obesity	2016	WHO	International
Action Plan for the Prevention and Control of Noncommunicable Diseases in the WHO European Region 2016–2025	2016	WHO Europe	Europe
Guideline: assessing and managing children at primary health-care facilities to prevent overweight and obesity in the context of the double burden of malnutrition	2017	WHO	International
Protect the progress: rise, refocus and recover	2020	WHO & UNICEF	International

IOTF, International Obesity Task Force; UNICEF, United Nations Children’s Fund; WHO, World Health Organization.

## Data Availability

Not applicable.
